# Novel quantitative parameters for pediatric dental arch morphology in children aged 4–12 years

**DOI:** 10.1186/s12903-026-08900-8

**Published:** 2026-06-18

**Authors:** Jiaqing Luo, Xiaobing Li

**Affiliations:** 1 School of Computer Science and Engineering, University of Electronic Science and Technology of China, No. 2006, Xiyuan Ave, West Hi-Tech Zone, Chengdu, Sichuan 611731 China; 2 State Key Laboratory of Oral Diseases, National Clinical Research Center for Oral Diseases, West China Hospital of Stomatology, Sichuan University, No. 14, People’s South Road, Chengdu, Sichuan 610041 China; 3 Department of Pediatric Dentistry, West China Hospital of Stomatology, Sichuan University, No. 14, People’s South Road, Chengdu, Sichuan 610041 China

**Keywords:** Quantitative parameters, Pediatric dental arch morphology, Digital dental models, Arch curve irregularity, Inter-arch coordination

## Abstract

**Objectives:**

This study aimed to develop novel quantitative parameters for pediatric dental arch morphology, providing a reproducible and interpretable approach for characterizing growth-related morphological patterns in children aged 4–12 years.

**Materials and methods:**

3 parameters were derived from digital dental models: Arch Curve Irregularity (ACI) quantified fine-scale alignment deviations based on differences between a spline-fitted occlusal curve and a smooth polynomial reference curve. Arch Shape Deviation (ASD) assessed large-scale arch form by comparison with population-based reference templates. Inter-Arch Shape Discrepancy (IASD) evaluated maxillary–mandibular coordination according to typical inter-arch coupling pattern. These parameters were applied to 473 digital models from children aged 4–12 years, including 274 normal occlusion and 199 malocclusion cases.

**Results:**

All parameters captured typical patterns of dental arch morphology while revealing substantial inter-individual variability. ACI represented the most frequently observed variation and often appeared in isolation. In contrast, isolated IASD findings were uncommon and were usually accompanied by deviations in arch shape or curvature. Combined parameter analysis revealed distinct configurations of large-scale, fine-scale, and inter-arch morphological variation that are not represented by conventional alignment-based indices. Longitudinal observations further distinguished transient alignment-related irregularities from more persistent morphological differences.

**Conclusions:**

These novel quantitative parameters provide a reproducible and interpretable approach for assessing pediatric dental arch morphology, capturing combined and persistent deviations from typical baselines. These parameters support longitudinal observation and comparative analyses of growth-related morphological patterns, offering a foundation for future research and potential clinical assessment.

## Introduction

Dental arch morphology undergoes continuous, age-specific changes during the critical developmental period of 4–12 years, spanning primary, mixed, and early permanent dentition. Characterizing these changes is challenging because arch form evolves dynamically with age and exhibits substantial individual variability, making subtle morphological differences difficult to capture. Existing evaluation approaches rely primarily on alignment-based indices or qualitative assessments, which offer limited insight into the multidimensional structure of dental arches.

Traditional orthodontic indices, such as the Peer Assessment Rating (PAR) index [1] and Little’s Irregularity Index [2], primarily quantify alignment-related features and provide limited characterization of overall dental arch morphology. In parallel, commonly used growth-related markers, including skeletal age and stature, have limited ability to capture the full complexity of dental development in pediatric populations. Longitudinal studies and age-stratified cross-sectional analyses have reported structured changes in dental arch dimensions across childhood, with coordinated variation in width, depth, and perimeter during key developmental periods [3–5]. Current research relies on group-level analyses and remains descriptive, without standardized quantitative tools to assess individual-level morphological deviations. As a result, a methodological gap persists in integrative, quantitative approaches for individualized assessment of pediatric dental arch morphology over developmental trajectories.

To address this gap, the aim of the present study is to develop a set of 3 complementary quantitative parameters for the reproducible and interpretable characterization of dental arch morphology in children aged 4–12 years.

1.  Arch Curve Irregularity (ACI) quantifies deviations of the occlusal curve from a smoothed reference curve. Aschheim et al. [6] proposed a mathematical framework using quadratic polynomial fitting and metric analysis to characterize morphological features of the maxillary anterior dental arch. Low-order polynomials (e.g., 4th- or 6th-order) capture the large-scale arch curvature [7], while higher-order splines (e.g., cubic Hermite splines) describe fine-scale variations [8]. ACI quantifies the difference between the 2 curves and provides a standardized numerical measure of fine-scale irregularity.
2.  Arch Shape Deviation (ASD) quantifies the extent to which an arch’s contour deviates from typical shape patterns. Previous morphometric studies, including our earlier work [4], have used polynomial curves and resampling methods to characterize such deviations. ASD scores are converted into deviation levels, enabling proportional and combined analysis with the other 2 parameters.
3.  Inter-Arch Shape Discrepancy (IASD) assesses the coordination between maxillary and mandibular arch shapes. Typical development shows strong alignment between the 2 arches, with high covariation in primary shape features such as depth, taper, and incisor position, as reported in a twin morphometric study [9]. Our previous work [10] further showed that mismatches in dental and alveolar contours were observed in a substantial proportion of malocclusion cases. IASD generalizes this concept by introducing a quantitative metric to assess deviations from typical maxillary–mandibular coupling.

Together, these 3 quantitative parameters provide a reproducible and interpretable approach for characterizing pediatric dental arch morphology. By relating individual digital models to typical developmental patterns, they capture complex morphological variation beyond conventional alignment-based indices and support longitudinal and comparative analyses of growth-related changes.

## Materials and methods

### Study sample

A total of 274 children (132 males, 142 females; age 4–12 years; mean ± SD: 7.38 ± 2.39 years) were selected from a community-based oral health survey of 2,695 school-age children conducted in Chengdu, China, between January 2021 and January 2023. Participants were included if they met all of the following criteria:

1. primary, mixed, or early permanent dentition;
2. Class I sagittal relationship, with a harmonious soft-tissue profile, approximately flat occlusal curves, minimal crowding (< 2 mm) or spacing (< 1 mm), and normal overjet (2–4 mm) and overbite (upper incisors covering < 1/3 of the lower incisors);
3. well-aligned arches with normal intercuspation;
4. intact dentition and physiologic tooth replacement;
5. symmetrical facial morphology consistent with normal growth patterns.

A total of 199 children with malocclusion (64 males, 135 females; age 5–12 years; mean ± SD: 8.19 ± 1.29 years) were recruited from the West China Hospital of Stomatology, Sichuan University. Participants were included if they met all of the following criteria:

1. mixed or early permanent dentition;
2. presence of malocclusion confirmed by lateral cephalometric analysis, including skeletal Class I (ANB 0°–5°), Class II (ANB > 5°), or Class III (ANB < 0°);presence of malocclusion confirmed by lateral cephalometric analysis, including skeletal Class I (ANB 0°–5°), Class II (ANB > 5°), or Class III (ANB < 0°);
3. transverse and/or sagittal dental arch deficiency, such as reduced arch width or shortened arch length.

Exclusion criteria were:

1. systemic or genetic disorders and upper airway disease;
2. previous orthodontic or prosthodontic treatment, or history of dentofacial surgery;
3. marked tooth wear, deformity, or structural defects;
4. incomplete or defective dental casts.
5. missing teeth due to agenesis (congenital absence of teeth).

### Digital model processing

Digital tooth models of the maxillary and mandibular dental arches were acquired using a UP3D laser scanner (UP400; 8 μm resolution). All scan data were exported as STL files and imported into MATLAB (R2024a) for normalization preprocessing. Each model was oriented to a consistent reference frame, and mesh noise was removed to ensure stable landmark detection.

Tooth segmentation used a curvature-guided surface segmentation method [11, 12]. Interproximal boundaries were identified from high-curvature areas and contour transitions, allowing each crown to be separated automatically. After segmentation, occlusal landmarks were identified by combining curvature peaks, height gradients, and fine-scale geometric features. The key anatomical reference points included the incisal edges of anterior teeth, canine cusps, and buccal cusps of premolars and molars. These landmarks were used to delineate the overall dental arch contour and morphological shape.

Landmarks were projected onto an occlusal plane aligned with each arch. A 6th-order polynomial curve was then fitted to represent the overall arch shape while reducing fine-scale noise. This order was selected based on prior literature and empirical performance, providing a balance between flexibility in capturing curvature and model stability without overfitting. The resulting curve served as a reference for each arch and formed the basis for calculating ACI, ASD, and IASD.

All measurements were generated using a standardized automated computational workflow with fixed parameters and identical processing procedures, with limited manual calibration where necessary.

### Arch curve irregularity (ACI)

Arch Curve Irregularity (ACI) is calculated by first fitting a 6th-order polynomial to capture the large-scale arch curvature, and then fitting a higher-order spline curve to capture fine-scale variations. In normal arches, the 2 fitted curves closely match, whereas in arches with increased fine-scale deviations (e.g., local alignment irregularities), the difference between the curves becomes more pronounced. The difference between the 2 curves is quantified using the Normalized Root Mean Square Error (NRMSE) [13]. The resulting NRMSE values are standardized as z-scores and mapped to deviation levels using predefined cutoffs: Typical (|z|≤1), Mild (1<|z|≤2), Moderate (2<|z|≤3), and Severe (|z|>3). The term “typical” is used to denote statistically representative morphological patterns within the reference distribution, whereas “normal” refers to clinically diagnosed occlusion status.

### Arch shape deviation (ASD)

Arch Shape Deviation (ASD) was quantified using a repeated clustering grading procedure. Following our previous work [4], each arch was first normalized and compared to a set of reference shapes via k-means clustering. Cluster centers derived from k-means clustering [10] served as reference templates. The distance of each arch to its nearest template was converted to a z-score and mapped to 4 deviation levels (Typical, Mild, Moderate, Severe). Larger distances reflect greater deviations in large-scale arch form, such as asymmetry, transverse discrepancies, or other shape-related variations. To account for variability, clustering and template matching were repeated 100 times with bootstrap perturbation, and the probability of each arch belonging to each deviation level was calculated. The final level was determined by the one with the highest assignment probability (i.e., the proportion of times it was selected across repetitions).

### Inter-arch shape discrepancy (IASD)

**Fig. 1:**
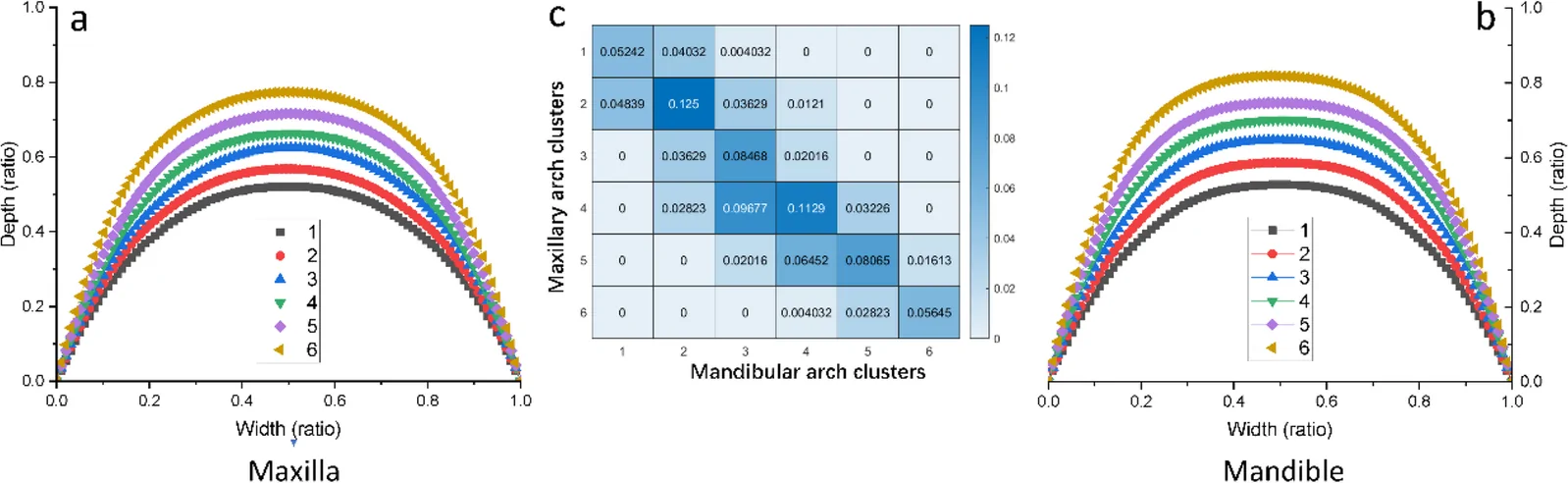
Templates (cluster centers) of maxillary (**a**) and mandibular (**b**) arch shapes and their cross-arch association matrix **c**. Numbers 1–6 denote cluster labels (shape groups). Each cell (*i*, *j*) in the heatmap shows the proportion of samples with typical occlusion assigned to maxillary cluster *i* and mandibular cluster *j*

Inter-Arch Shape Discrepancy (IASD) was calculated by normalizing the maxillary and mandibular dental arches and assigning each arch to a shape category using k-means clustering. To capture variability, the clustering was repeated 100 times using bootstrap resampling. For each repetition, the association between the paired maxillary and mandibular arches was measured. IASD was derived from the cross-arch association matrix, in which maxillary–mandibular shape pairings that were rare in the typical reference cohort were considered atypical. Figure 1 shows the templates (cluster centers) and the reference associations used to compute IASD.

### Integration of parameters

Taken together, ACI, ASD, and IASD constitute a set of complementary, reference-based quantitative parameters for the assessment of dental arch morphology. These parameters support proportion-based and combined-parameter analyses, providing reproducible and interpretable measures of morphological variation relative to typical developmental patterns over time.

## Results

### Arch curve assessment and irregularity

**Fig. 2:**
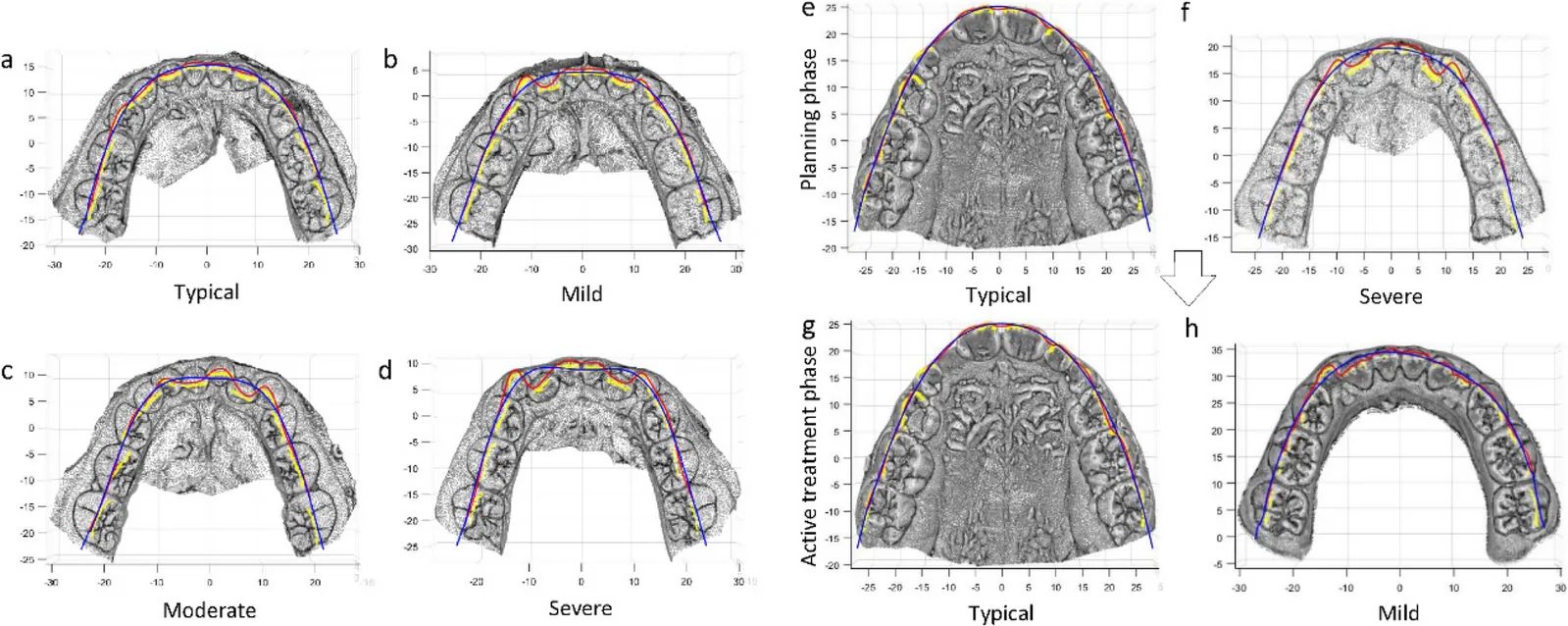
Representative examples of 4 irregularity levels (**a**–**d**) and longitudinal changes over time **e**–**h**. Yellow dots mark the occlusal landmarks; red lines show the spline-fitted curves; blue lines show the polynomial reference curves. Greater separation between the red and blue curves indicates a higher degree of irregularity

Increasing ACI grades corresponded to greater divergence between the spline and polynomial fits. The magnitude of this deviation, expressed as the NRMSE, was used to classify curve irregularity into 4 levels (Fig. 2 (a-d)). These levels clearly illustrate the evolution from almost indistinguishable curves to significant fine-scale deviations from the large-scale arch shape. Representative cases demonstrated longitudinal changes in measured deviation across different time points. In some cases, the spline and polynomial curves became closer over time, indicating a change in arch morphology relative to the reference curves. For example, a change from Severe to Mild in mandibular arch irregularity was observed between pre- and post-treatment measurements (Fig. 2 (f, h)).

### Arch shape patterns and deviation

**Fig. 3:**
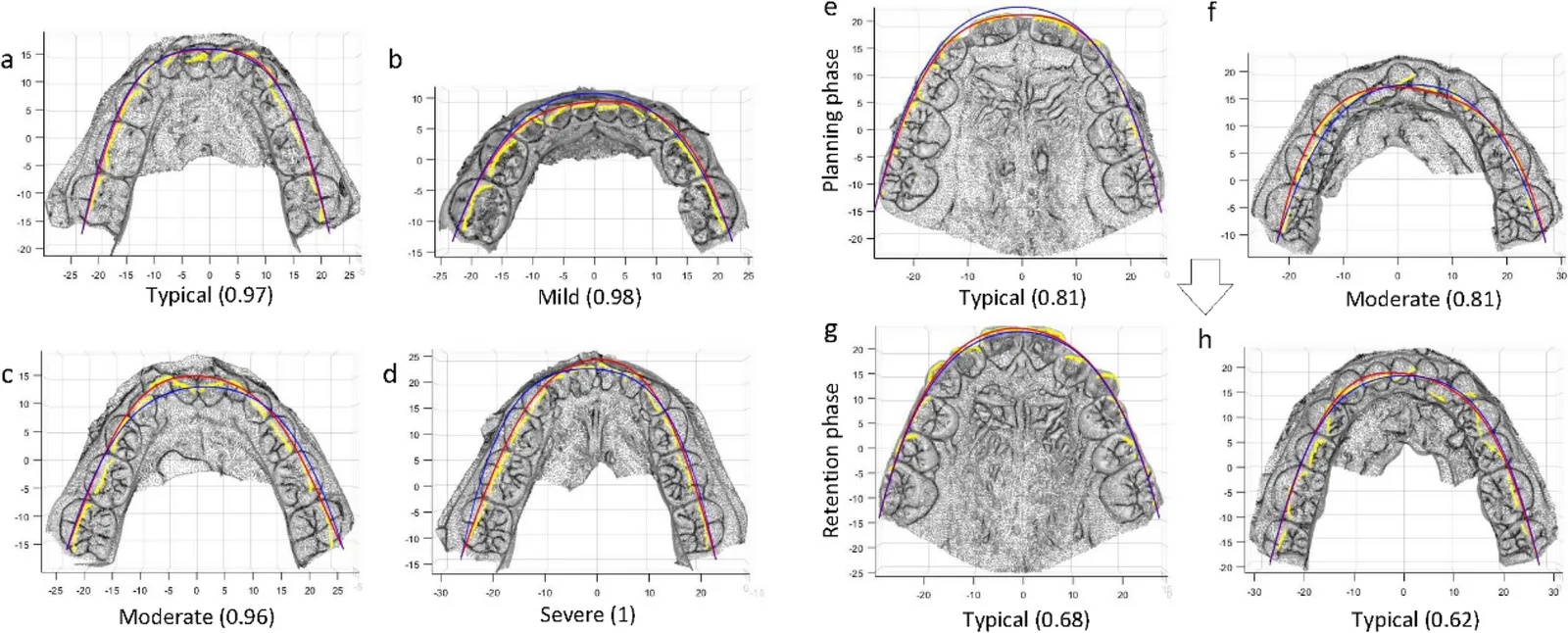
Representative examples of arch shape deviation (ASD) levels and longitudinal changes. Examples of the 4 ASD deviation levels (**a**–**d**), visualized with yellow occlusal landmarks, blue reference arch templates, and red polynomial-fitted curves. Longitudinal comparisons of maxillary (**e**, **g**) and mandibular (**f**, **h**) arches. Values in parentheses represent the probability of belonging to the assigned deviation level

The ASD grading results demonstrated clear distinctions among the 4 deviation levels (Typical, Mild, Moderate, Severe), as illustrated in Fig. 3 (a–d). Increasing ASD deviation was visually associated with greater divergence between the fitted polynomial curve (individualized dental arch shape) and the reference arch template (typical reference arch shape).

A representative treatment case is shown in Fig. 3 (e–h). The maxillary arch remained Typical (Fig. 3 (e, g)), while the mandibular arch changed from Moderate (0.81) to Typical (0.62) (Fig. 3 (f, h)).

### Maxillary–mandibular coordination

Inter-arch shape relationships showed substantial variability across the sample. For each sample, bootstrap resampling was repeated, and IASD probability was calculated as the proportion of times the maxillary and mandibular arches fell outside the typical association. Higher IASD probabilities indicate a stronger and more consistent deviation from typical inter-arch coordination.

**Fig. 4:**
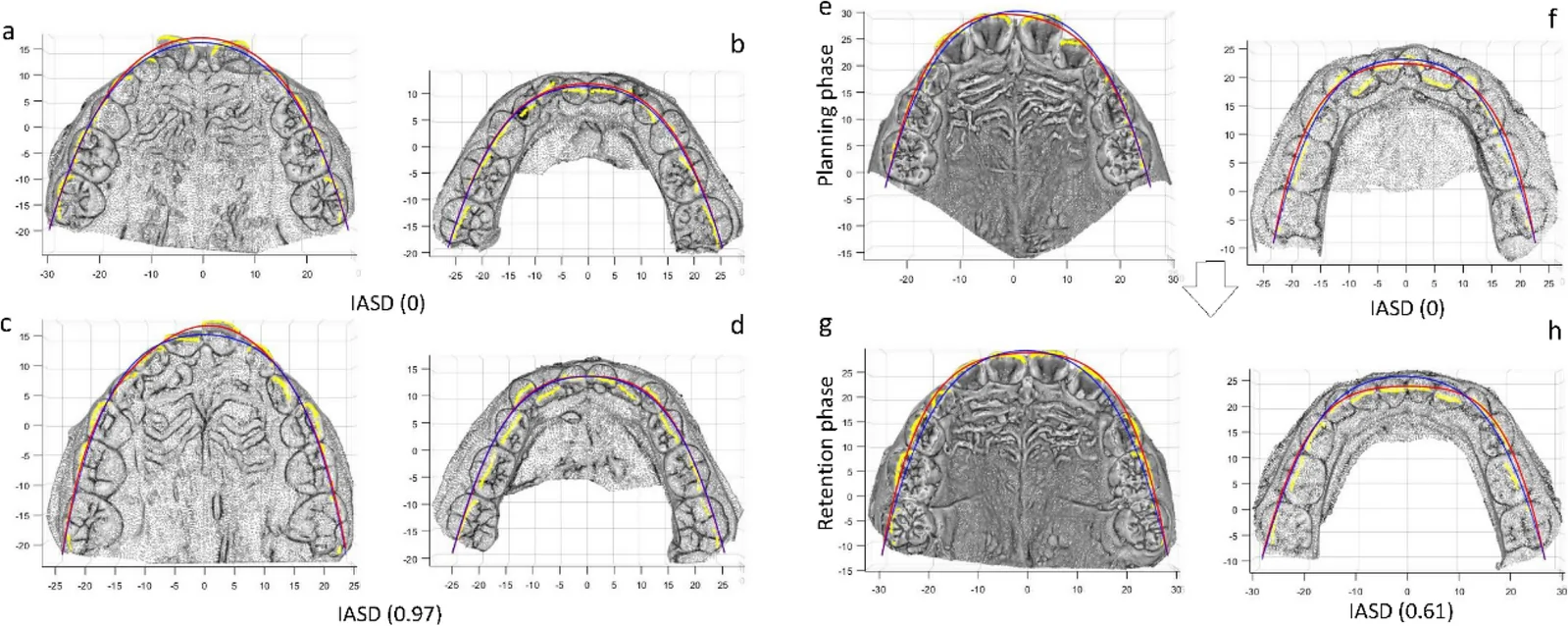
Representative examples of maxillary–mandibular coordination based on IASD probability. Paired arches with low and high IASD probabilities (**a**–**d**), illustrating well-matched versus mismatched arch shapes. Additional paired examples at different observation points (**e**–**h**) demonstrate changes in IASD probability over time. Yellow dots mark occlusal landmarks; blue and red curves represent the reference arch templates and polynomial-fitted curves, respectively

Most samples demonstrated very low IASD probabilities (usually close to 0), indicating stable maxillary–mandibular coordination (Fig. 3 (a, b)). Only a few samples exhibited high IASD probabilities approaching 1 (Fig. 4 (c, d)), corresponding to significant inter-arch discrepancy. Notably, IASD probability increased from near 0 pre-treatment to 0.61 post-treatment in a representative case, indicating the emergence of measurable inter-arch discrepancy (Fig. 4 (e-h)).

### Integrated pattern analysis

An integrated analysis was conducted by classifying each parameter as typical or atypical based on predefined thresholds. For Arch Curve Irregularity (ACI) and Arch Shape Deviation (ASD), a z-score threshold of 1.96 was applied as a conventional statistical criterion corresponding to the 95% reference range under a standard normal distribution. This cutoff was used in an exploratory manner to categorize morphological deviation in the present morphometric analysis. A deviation was recorded when either the maxillary or mandibular arch exceeded this threshold. For Inter-Arch Shape Discrepancy (IASD), atypical was defined as an IASD probability greater than 0.05.

**Fig. 5:**
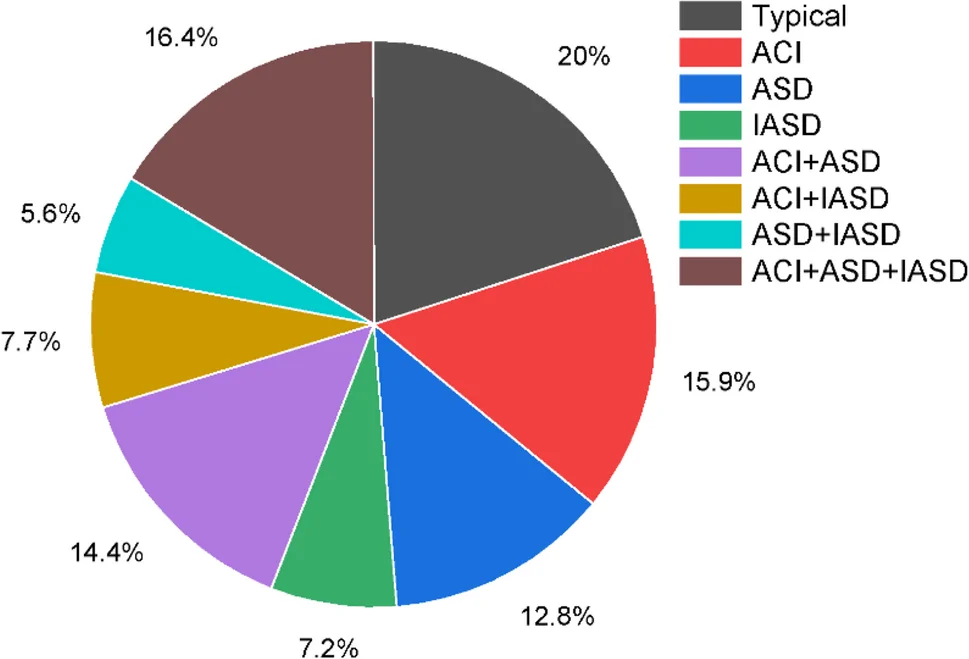
Integrated distribution of atypical patterns across 3 parameters. Pie chart showing the proportions of samples classified by combined status of Arch Curve Irregularity (ACI), Arch Shape Deviation (ASD), and Inter-Arch Shape Discrepancy (IASD). Each sector represents one of the 8 possible combinations of typical or atypical parameter values

Figure 5 summarizes the distribution of subjects across the 8 possible combinations of the 3 parameters. The largest proportion of the cohort (20.0%) showed typical morphology across all 3 parameters. Concurrent deviations across all 3 parameters were also common, accounting for 16.4% of samples, reflecting complex deviations involving arch curvature, arch shape, and inter-arch coordination.

For isolated parameters, atypical ACI was the most frequent (15.9%), followed by atypical ASD (12.8%). Isolated atypical IASD was less frequent (7.2%), suggesting that inter-arch discrepancy more often co-occurred with deviation in arch form or curvature rather than presenting alone.

The association between parameter classification (typical/atypical) and clinically diagnosed malocclusion was evaluated using Fisher’s exact test and Cramer’s V coefficient. All 3 parameters were significantly associated with malocclusion (all *P* < 0.001). ACI showed a strong association (Cramer’s V = 0.6), whereas ASD (V = 0.26) and IASD (V = 0.16) presented moderate-to-weak associations. These results support the ability of the proposed parameters to quantify morphological variation associated with clinical occlusion status.

## Discussion

ACI, ASD, and IASD provide independent quantitative characterization of fine-scale alignment, large-scale arch shape, and inter-arch coordination, respectively. Dental arch development shows consistent reference patterns but substantial inter-individual variability, with alignment irregularities often transient during growth [3, 14], supporting individualized morphological description relative to reference patterns.

Arch length increases progressively, while transverse dimensions stabilize earlier [15]. Untreated cohorts have also demonstrated strong covariation between maxillary and mandibular arch shapes [16]. The IASD probability quantifies this inter-arch coordination, enabling comparison of individual digital models with reference patterns to describe variations in morphological development.

Compared with conventional indices (PAR, ABO Discrepancy Index, Little’s Irregularity Index) [1, 2, 17], these parameters provide complementary information on arch form and developmental variation. Little’s Irregularity Index quantifies only the linear displacement of the 6 anterior teeth, which may not fully represent broader morphological deviations. ACI quantifies fine-scale alignment deviations, while ASD captures large-scale arch shape differences, consistent with morphometric approaches applied to digital dental models [10, 18].

Morphological variations during mixed dentition have been associated with subsequent changes in arch form and inter-arch coordination [15, 19]. In this context, ASD and IASD values reflect the degree of variation in arch shape and inter-arch coordination, with higher values indicating more pronounced deviations and lower values corresponding to subtler or transient alignment variations. Based on reference to typical developmental patterns, ACI, ASD, and IASD can help distinguish mild or transient variations from more complex combined patterns. In this cohort, mild atypical ACI values were relatively common, whereas isolated atypical IASD values were rare; concurrent atypical patterns across parameters were more frequently observed, suggesting coordinated maxillary–mandibular developmental variation, which is consistent with previous reports describing coordinated arch development [3, 20].

The proposed morphometric parameters were designed as quantitative descriptors of structural deviation patterns rather than standalone diagnostic tools. Nevertheless, their associations with clinically diagnosed malocclusion suggest potential utility as supplementary information for clinical assessment. Parameters with stronger associations, such as ACI, may correspond to morphological features that are more readily identified in routine examination, whereas weaker associations, particularly IASD, may represent subtler structural variations that are less easily perceived and therefore potentially overlooked in conventional clinical evaluation. The reliability and reproducibility of digital model–based measurements further support their applicability for longitudinal morphological assessment [18, 21].

This study has several limitations. Validation of the proposed parameters against broader malocclusion phenotypes warrants further investigation. Methodological choices, including polynomial order, spline fitting, and template construction, could be further refined as larger datasets become available, consistent with recent work in geometric morphometrics [10, 22]. The deviation threshold applied in this study (|z|>1.96) was exploratory and may require further adjustment for research applications. The unbalanced sex distribution between the normal and malocclusion groups should be acknowledged. Although sexual dimorphism is relatively limited before the pubertal growth spurt, the unequal sex distribution may still introduce potential demographic bias. Future studies that integrate dental arch morphometrics with skeletal and soft-tissue developmental measures may enhance comparative analyses. In addition, larger and multi-center cohorts are needed to confirm the generalizability of these parameters across different populations and age ranges.

## Conclusion

This study introduces 3 interpretable quantitative parameters (ACI, ASD, IASD) for pediatric dental arch morphology assessment. By converting complex 3D dental model data and experience-based observations into standardized deviation measures, these parameters offer a structured, reproducible, and interpretable approach to characterize morphological variation relative to typical developmental patterns. The proposed parameters provide a methodological foundation for longitudinal, comparative, and developmental studies, supporting objective analysis of dental arch morphology, with potential to inform future research and clinical assessment.

## Data Availability

The data of this study are available from the corresponding authors upon reasonable request.

## Abbreviations

ACI: Arch Curve Irregularity
ASD: Arch Shape Deviation
IASD: Inter-Arch Shape Discrepancy
PAR: Peer Assessment Rating
ANB: a specific measurement made on lateral cephalograms. It stands for A-point, Nasion, B-point. These points are landmarks on the X-ray image that correspond to specific anatomical structures
NRMSE: Normalized Root Mean Square Error
